# Supplementary material for: Tuning the Surface Structure of Polyamide Membranes Using Porous Carbon Nitride Nanoparticles for High-Performance Seawater Desalination
Source: Membranes (Basel). 2020 Jul 24;10(8):163. doi: 10.3390/membranes10080163 (PMC7466004; doi:10.3390/membranes10080163)
Supplement: Supplementary file 1 [file membranes-10-00163-s001.pdf]

membranes

Supporting Information

Tuning the Surface Structure of Polyamide Membranes Using Porous Carbon Nitride Nanoparticles for High-Performance Seawater Desalination

Zongyao Zhou †, Xiang Li †, Digambar B. Shinde, Guan Sheng, Dongwei Lu, Peipei Li and Zhiping Lai *

Advanced Membranes and Porous Materials Center, Division of Physical Science and Engineering, King Abdullah University of Science and Technology (KAUST), Thuwal 23955-6900, Saudi Arabia
Zongyao.zhou@kaust.edu.sa (Z.Z.); Xiang.li@kaust.edu.sa (X.L.); Digambar.Shinde@kaust.edu.sa (D.B.S.); Guan.sheng@kaust.edu.sa (G.S.); dongwei.lu@kaust.edu.sa (D.L.); peipei.li@kaust.edu.sa (P.L.)
* Correspondence: Zhiping.lai@kaust.edu.sa
† These authors contribute equally.

Supplementary Figures

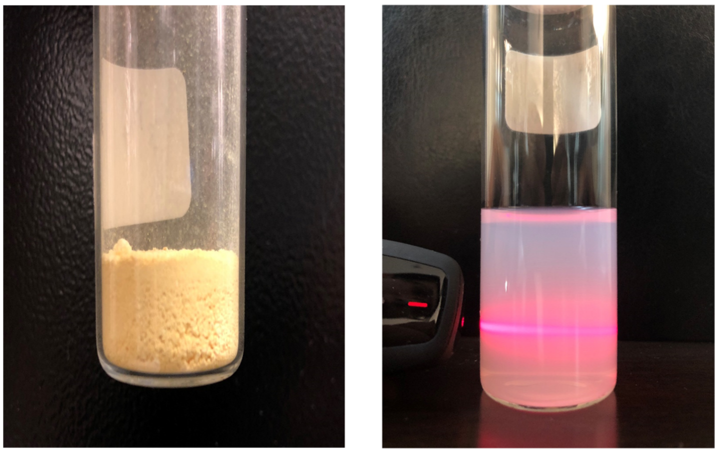

Figure S1. Optical photographs of C_3N_4 powders and the aqueous solution.

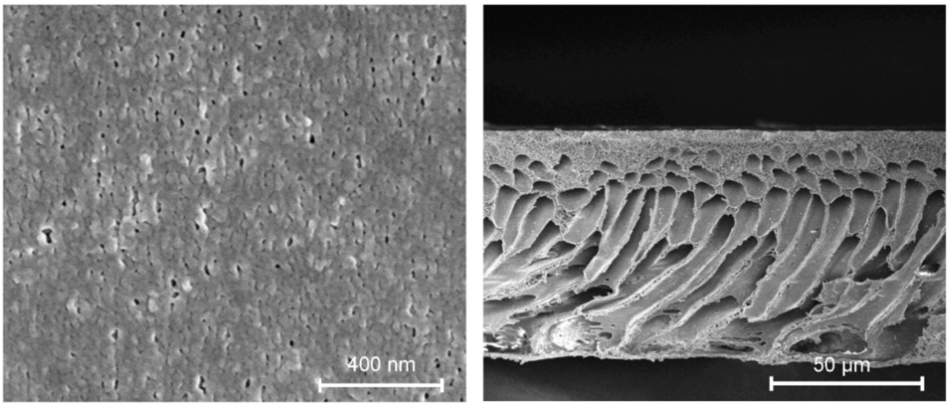

Figure S2. SEM images of the prepared PSf substrate.

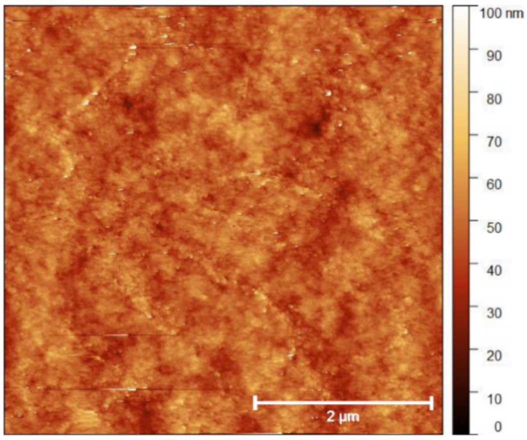

Figure S3. AFM image of the prepared PSf substrate.

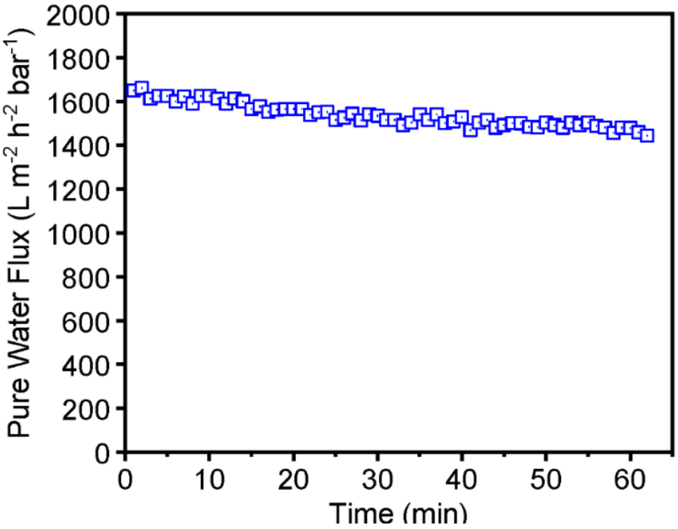

Figure S4. Pure water flux of the prepared PSf substrate.

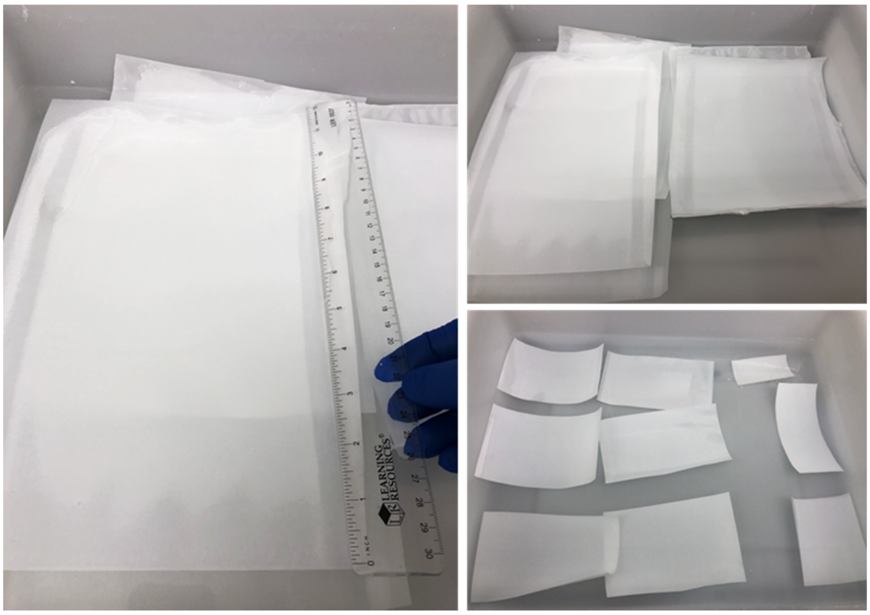

Figure S5. Optical photographs of the prepared pristine TFC and TFN membranes.

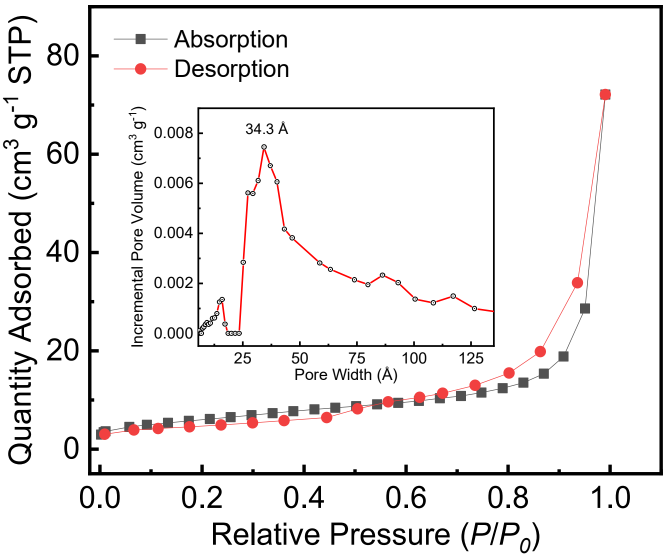

Figure S6. N_2 adsorption isotherm (G), and pore size distribution (G inset) of the prepared C_3N_4.

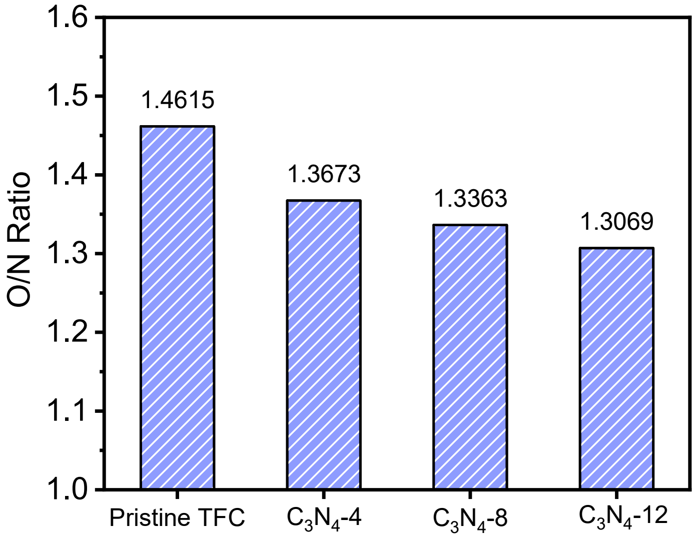

Figure S7. O/N ration of PA layer fabricated with various amounts C_3N_4 nanosheets. The degree of cross-linking was calculated by $\frac{X}{X+Y} \times 100\%$, where X and Y were calculated from the following equations, 3X+4Y = O1s and 3X+2Y=N1s.

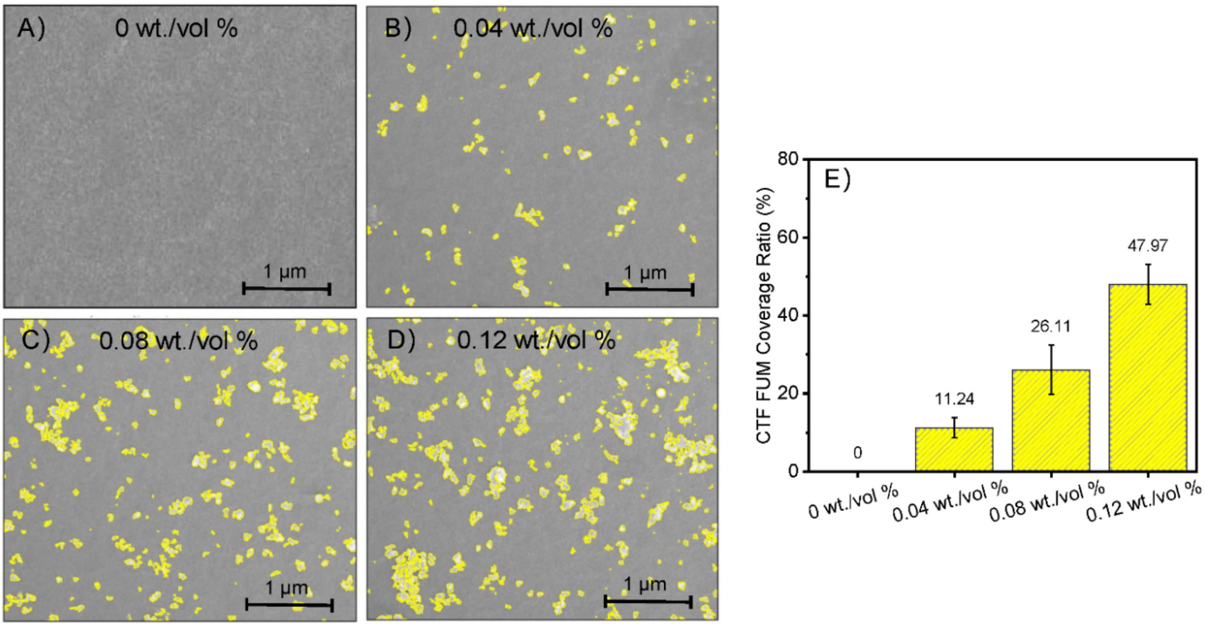

Figure S8. SEM images of C_3N_4 deposition on PSf support and surface coverage of the deposited particles on supports, as measured by *Image J* software.

We supposed that the distribution of C_3N_4 on PSf substrate after removing the excess aqueous solution has a great effect on the next step of interfacial polymerization. As shown in Fig. S8, C_3N_4 uniformly distributed on PSf support surface benefitting from their excellent dispersion in water and suitable particle size. The surface coverage ratio of the deposited C_3N_4 on the PSf surface obviously increased from 11.24% to 47.97% as the increased concentration of the particles in MPD solution from 0.04 wt./vol % (C_3N_4-4) to 0.12 wt./vol % (C_3N_4-12). Note that with increasing the C_3N_4 loading up to 0.12 wt./vol%, most of the nanoparticles were deposited well on PSf surface as a monolayer but some overlapped forming agglomeration. The interesting deposition features of the nanosheets indicate that an appropriate nanoparticles loading amount is significant.

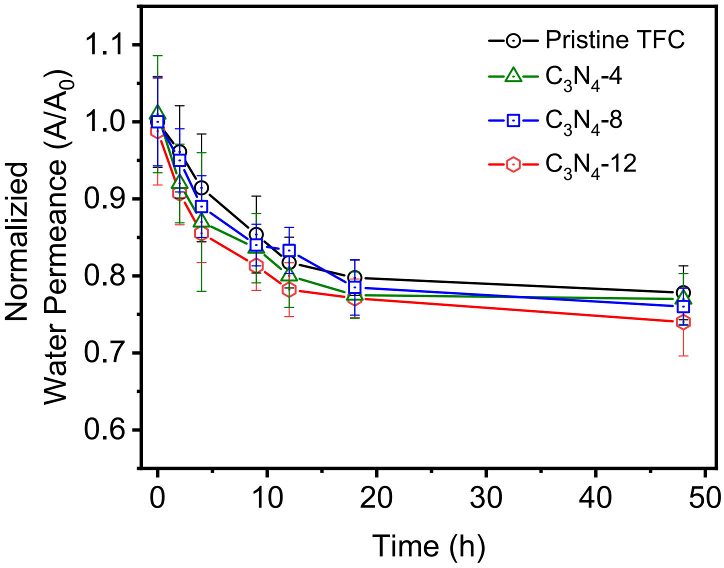

Figure S9. Normalized water permeance under long-term operation.

Supplimentary Tables

Table S1. Elementary composition of PA layer with various C_3N_4 amount.

RO Membranes	C1s [%]	O1s [%]	N1s [%]
Pristine TFC	77.6	13.3	9.1
C3N4-4	76.1	13.8	10.1
C3N4-8	77.1	13.1	9.8
C3N4-12	76.7	13.2	10.1

Table S2. *Jw*, *B* and *R* of TFC and TFN membranes.

RO Membranes	J_w [LMH/bar]	B [LMH]	R [%]
Pristine TFC	1.7 ± 0.4	0.5 ± 0.1	98.0 ± 0.4
C3N4-4	2.8 ± 0.2	0.3 ± 0.1	99.2 ± 0.3
C3N4-8	3.2 ± 0.2	0.2 ± 0.1	99.5 ± 0.2
C3N4-12	3.6 ± 0.2	1.2 ± 0.2	97.6 ± 0.4

Table S3. Comparison of the *A*, *B* and *R* of TFC and TFN membranes reported in references and in this work.

RO Membranes	ΔP [bar]	NaCl [ppm]	Jw [LMH/bar]	B [LMH]	R [%]	Ref.
PA-TFC	15.5	2000	1.7 ± 0.4	0.5 ± 0.1	98.0 ± 0.4	This work
C3N4-4	15.5	2000	2.8 ± 0.2	0.3 ± 0.1	99.2 ± 0.3	This work
C3N4-8	15.5	2000	3.2 ± 0.2	0.2 ± 0.1	99.5 ± 0.2	This work
C3N4-12	15.5	2000	3.6 ± 0.2	1.2 ± 0.2	97.6 ± 0.4	This work
Dow-SW30HR	15.5	2000	0.92 ± 0.12	0.03 ± 0.01	99.3 ± 0.1	1
Dow-BW30	15.5	2000	3.77 ± 0.13	0.53 ± 0.06	96.8 ± 0.3	1
Sepro-RO1	15.5	2000	4.90 ± 0.11	0.59	99.1 ± 0.1	2
Sepro-RO4	15.5	2000	0.97 ± 0.02	0.16	98.8 ± 0.1	2
TFN-ZIF-8	15.5	2000	3.35 ± 0.08	0.22	98.5 ± 0.5	2
TFC-1-GO	15.5	2000	5.42 ± 0.28	1.32	98.2 ± 0.7	3
TFC GO	15.5	2000	1.97	0.17	98	4
0.5 wt.% o-CNT TFC	15.5	2000	3.03	0.31	97.7	5
ASP-Silica RO	15.5	2000	4.16	2.07	96.4	6

References

1. Lee, J.; Wang, R.; Bae, T.-H., High-performance reverse osmosis membranes fabricated on highly porous microstructured supports. *Desalination* **2018**, *436*, 48–55.
2. Duan, J.; Pan, Y.; Pacheco, F.; Litwiller, E.; Lai, Z.; Pinnau, I., High-performance polyamide thin-film-nanocomposite reverse osmosis membranes containing hydrophobic zeolitic imidazolate framework-8. *J. Membr. Sci.* **2015**, *476*, 303–310.
3. Lee, J.; Jang, J. H.; Chae, H.-R.; Lee, S. H.; Lee, C.-H.; Park, P.-K.; Won, Y.-J.; Kim, I.-C., A facile route to enhance the water flux of a thin-film composite reverse osmosis membrane: incorporating thickness-controlled graphene oxide into a highly porous support layer. *J. Mater. Chem. A* **2015**, 3,, 22053–22060.
4. Ali, M. E. A.; Wang, L.; Wang, X.; Feng, X., Thin film composite membranes embedded with graphene oxide for water desalination. *Desalination* **2016**, *386*, 67–76.
5. Lee, T. H.; Lee, M. Y.; Lee, H. D.; Roh, J. S.; Kim, H. W.; Park, H. B., Highly porous carbon nanotube/polysulfone nanocomposite supports for high-flux polyamide reverse osmosis membranes. *J. Membr. Sci.* **2017**, *539*, 441–450.
6. Li, Q.; Yu, H.; Wu, F.; Song, J.; Pan, X.; Zhang, M., Fabrication of semi-aromatic polyamide/spherical mesoporous silica nanocomposite reverse osmosis membrane with superior permeability. *Appl. Surf. Sci.* **2016**, *363*, 338–345.
